# Using program evaluation to support knowledge translation in an interprofessional primary care team: a case study

**DOI:** 10.1186/s12875-016-0538-4

**Published:** 2016-10-06

**Authors:** Catherine Donnelly, Lyn Shulha, Don Klinger, Lori Letts

**Affiliations:** 1 School of Rehabilitation Therapy, Queen’s University, 31 George Street, Kingston, ON K7L 4B4 Canada; 2 Faculty of Education, Queen’s University, Duncan McArthur Hall, 511 Union Street, Kingston, ON K7M 5R7 Canada; 3 School of Rehabilitation Science, McMaster University, Room 403, 1400 Main St. W., Hamilton, ON L8S 1C7 Canada

**Keywords:** Interdisciplinary health team, Primary health care, Knowledge translation, Integrated knowledge translation, Program evaluation, Case study

## Abstract

**Background:**

Evaluation is a fundamental component in building quality primary care and is ideally situated to support individual, team and organizational learning by offering an accessible form of participatory inquiry. The evaluation literature has begun to recognize the unique features of KT evaluations and has described attributes to consider when evaluating KT activities. While both disciplines have focused on the evaluation of KT activities neither has explored the role of evaluation in KT. The purpose of the paper is to examine how participation in program evaluation can support KT in a primary care setting.

**Methods:**

A mixed methods case study design was used, where evaluation was conceptualized as a change process and intervention. A Memory Clinic at an interprofessional primary care clinic was the setting in which the study was conducted. An evaluation framework, Pathways of Influence provided the theoretical foundation to understand how program evaluation can facilitate the translation of knowledge at the level of the individual, inter-personal (Memory Clinic team) and the organization. Data collection included questionnaires, interviews, evaluation log and document analysis. Questionnaires and interviews were administered both before and after the evaluation: Pattern matching was used to analyze the data based on predetermined propositions.

**Results:**

Individuals gained program knowledge that resulted in changes to both individual and program practices. One of the key themes was the importance clinicians placed on local, program based knowledge. The evaluation had less influence on the broader health organization.

**Conclusions:**

Program evaluation facilitated individual, team and organizational learning. The use of evaluation to support KT is ideally suited to a primary care setting by offering relevant and applicable knowledge to primary care team members while being sensitive to local context.

**Electronic supplementary material:**

The online version of this article (doi:10.1186/s12875-016-0538-4) contains supplementary material, which is available to authorized users.

## Background

It has been recognized that primary care has unique issues related to knowledge translation (KT). As the first point of contact with the health care system, health issues may not be clearly articulated and broad health services are provided to a range of conditions across the lifespan [1]. Primary care clinicians have been found to rely heavily on clinical practice guidelines however these are primarily developed for single diseases, “filtered by specialists” making them difficult to apply to the primary care setting where patients often present with multiple chronic conditions [1, 2]. Menar and colleagues explored KT and primary care, and stressed the importance of adopting an integrated knowledge translation (IKT) approach within primary care as a way to actively engage primary care providers in the research process and support the production of contextually relevant knowledge [1]. Integrated knowledge translation (IKT) is the term used to describe the active collaboration between researchers and research users in all parts of the research process [3].

The Knowledge to Action Framework is a model for conceptualizing the movement of knowledge into practice and has been adopted by the Canadian Institute of Health Research (CIHR) [3]. The KTA framework is divided into two components: knowledge creation and action. The Action phase represents activities used to assist in the application of knowledge, with eight specific processes including the evaluation of outcomes. As the framework highlights, both the KT and program evaluation literature have focused on the evaluation of KT intervention [4–7] but neither has explored how engaging in a program evaluation can support KT.

### Program evaluation and knowledge translation

Program evaluation can be differentiated from research by its central focus on practice driven questions and goals of program and organizational improvement [8]. In primary care terms such as continuous quality improvement (CQI) and quality frameworks are often used when referring to processes that ensure quality care and outcomes. Program evaluation has many similarities to CQI, and some view them within a continuum of approaches to support organizations and program delivery. CQI is specifically focused on processes and systems, and focuses on ongoing improvements to deliver quality outcomes [9]. Program evaluation on the other hand is a broader concept and includes a wide range of approaches (e.g. summative, formative) and whose goal is ultimately aimed at determining the merit and worth of programs [10].

### Participatory inquiry and evaluation

In the many forms of program evaluation, stakeholder participation is purposefully cultivated to facilitate learning and knowledge building [11–15]. These practices reflect early and ongoing research on how participation enhances relevancy and therefore stakeholder use of evaluations [14, 15]. While IKT is often described as a process akin to participatory research, evaluation also seeks to engage participants in the evaluation process [12]. Given the emphasis on both KT and quality improvement initiatives in primary care [1, 16, 17] understanding evaluation’s role in KT can provide valuable insights into how evaluation can be structured to support both the creation of knowledge that addresses local practice needs and the application of this knowledge to enhance practice outcomes.

Evaluation theorists Henry and Mark [18, 19] offer a framework that can explore how evaluation can be used to support KT. Mark and Henry’s framework examines the influence of evaluation at the level of the individual, interpersonal and organization, where evaluation is conceptualized as change process. Viewing evaluation as a change process considers the engagement in the evaluation as a process that supports the application of new knowledge and research to practice. A recent systematic review of KT interventions in primary care highlighted the need to consider context and ensure interventions are applicable to the specific primary care settings [20]. Given evaluations focus on program-based questions, evaluation it ideally situated to be considered an approach that can support the uptake of research to practice in primary care. To date there has been no exploration of how evaluation can be used to support KT in primary care [20]. This study seeks to answer the following questions: How does participation in an evaluation influence a) individual members in the program, b) interpersonal behaviours in the program, and c) the broader primary care organization.

## Methods

A prospective, multiple methods case study design was employed [21, 22]. Case study research focuses on understanding a given phenomenon in a real-life environment and involves the collection of detailed information using a variety of data sources [22]. Evaluation has been used to examine the impact of KT activities, but there have been no records of how program evaluation as a process can be formally used to support KT in primary care. A case study design offered a methodology to gain an in-depth understanding of if, how, and why evaluation can be used for KT. The Pathways of Influence [18, 19] was the theoretical framework used to demonstrate how evaluation can support both knowledge creation and its application to practice during one evaluation in a primary care setting.

Ethics approval was provided by the Queen’s University Health Sciences and Affiliated Teaching Hospitals Research Ethics Board (HSREB) (approval #6006766). Each participant provided written and informed consent to participate in the study and were made aware that results from the study would be disseminated and published.

### Case study context

An evaluation of a Memory Clinic at an interprofessional primary care organization in the province of Ontario, Canada provided the context for the study. The Memory Clinic was part of an informal group of primary-care based memory clinics within the province of Ontario, Canada. Prior to the implementation of the Memory Clinic, all members completed a formal training to gain knowledge in the area of dementia. With long wait times to access specialist services, the objectives of the Memory Clinic were to facilitate the early diagnosis of memory disorders and provide community and caregiver support in a primary care context. Patients and caregivers attended a 2-hour interprofessional assessment. Following the assessment, a diagnosis was made and an individual care plan was provided. The Memory Clinic was offered on a monthly basis to patients with memory impairments and their families and was delivered by an interprofessional team of health providers including two physicians, two nurses, an occupational therapist, a social worker, a community pharmacist and an Alzheimer Society representative [23].

### Evaluation approach

The evaluation used a participatory approach [11, 24] and was also informed by efforts to support a knowledge translation approach to evaluation [25]. *The Program Evaluation Standards* [10] provided a foundation to conduct an ethical and quality evaluation. The intention of bringing these approaches together was to orchestrate a quality and collaborative evaluation that facilitated the development and refinement of the Memory Clinic through the ongoing translation of research and evaluation data. The study used a novel approach to evaluation, developed specifically to support KT in primary care, which was termed a KT-informed evaluation [25]. The KT-informed evaluation was designed to be intentional in facilitating the application of emerging evaluation knowledge into practice and attended to the empirical evidence (original studies or synthesized knowledge) that grounded the program and the clinicians within the program. The evaluation was cognizant of how empirical and formalized knowledge informed each phase of the evaluation: (a) ensuring evaluation questions were informed both by context and external evidence, and (b) that emerging and final findings were considered in light of current research. Three intentional activities were included in this approach; weekly e-newsletters, monthly Evaluation Process Meeting to review and discuss emerging findings and an evaluator presence in the program. A detailed description of this approach has been described in the literature [25].

#### Participatory evaluation

This evaluation was designed to support KT by adopting a participatory approach. Participatory evaluation involves some degree of collaboration between those conducting the evaluation and the stakeholders [11].

The extent to which an evaluation is participatory can be determined by mapping the evaluation process onto three dimensions of collaborative inquiry [11]; control of technical evaluation decisions, diversity of stakeholders selected for participation and depth of participation. Each dimension was considered in the evaluation design. In this evaluation, the evaluator ultimately led the technical evaluation decisions, with strong input obtained from program members at all stages throughout the evaluation. All organizational stakeholders were represented in the Evaluation Committee, whose membership included Memory Clinic clinicians, along with the Alzheimer society representative and the organization’s Executive Director; providing clinical, community and administrative perspectives. Members participated in the evaluation through monthly Evaluation Process Meetings and email communication; offering feedback and input into all aspects of the evaluation including the design, interpretation of data and translation of findings into the program.

### Participants

As the goal of this paper is to examine how evaluation can support KT, the evaluation of the Memory Clinic was the focus of the case study. All members of the Memory Clinic sat on the Memory Clinic’s Evaluation Committee and each member was invited to participate in the study. Six of the seven original Evaluation Committee members agreed to participate at the outset of the study. One member who did not participate at intake agreed to do so at follow-up. Due to incomplete data, questionnaires from the seventh participant were not included in the analysis, however a follow-up interview was conducted and included in the study. Two additional members joined the Memory Clinic over the course of the 8-month evaluation, but were not included in the sample.

### Data collection

Table 1 provides a summary of the overall data collection tools and timing of administration. Multiple sources of data were collected over 11-months, at three points in time.

**Table 1:** Data collection timeline

Data collection	Pre	Post	Follow-up
Edmonton research orientation scale	•	•	
	•	•	
Memory clinic knowledge questionnaire	•	•	
Interview – understanding individual and interactive levels	•	•	
Interview – understanding the collective level			•
Program documents			
Evaluation log			

All questionnaires were printed and paper copies were provided to participants, along with a self-addressed, stamped envelope. Each participant completed the questionnaires individually at their convenience and returned these via mail, to the primary author.

#### Edmonton research orientation survey

The Edmonton Research Orientation Survey [26] is a self-report tool that asks participants about their attitudes toward research and about their potential to use research findings. The assessment contains 38 items with four subscales. The items are scored on a 5-point Likert scale from strongly disagree to strongly agree, with higher scores indicating a more positive research orientation. The Edmonton Research Orientation Survey (EROS) was developed in the context of rehabilitation and has been used across health disciplines, including nursing [27–29]. The EROS has demonstrated internal consistency reliability (Cronbach’s alpha =0.83–0.89) and content and concurrent validity in rehabilitation, nursing and general hospital settings [30]. This is the first known use within a primary care setting.

#### Collaborative practice assessment tool

The Collaborative Practice Assessment Tool (CPAT) is a self-report questionnaire designed to assess individual team members’ perceptions of collaborative practice. The CPAT contains 56 items across eight domains that have been identified in the literature as relating to interprofessional collaborative practice. Results of two pilot tests have demonstrated that the CPAT is a valid and reliable tool [31].

#### Memory clinical knowledge questionnaire

The Memory Clinical Knowledge Questionnaire (MCK) was developed for this study by the authors to assess participant’s knowledge about assessment and intervention practices related to memory. No similar measures were found in the literature. The MCK questionnaire consisted of 6 close-ended and 3 open-ended questions asking respondents about their confidence and breadth of knowledge related to memory disorders as well as memory assessments and interventions currently used. See Additional file 1: Memory Clinic Knowledge Questionnaire.

#### Interviews

Fourteen interviews were conducted with participants from the Evaluation Committee. Interviews lasted between 20 and 60 min. Questions were developed by the research team and guided by Mark and Henry’s Pathways of Influence framework [18, 19] Interviews were conducted with all participants before and after the evaluation and held in a quiet, private office. Participants were asked how the evaluation influenced individuals’ clinical practices, knowledge and attitudes. Questions were also asked about how the evaluation influenced interactions with team members, patients and other knowledge networks. Three month follow-up interviews were conducted with two individuals identified to have influence at the level of the broader primary care organization.

#### Evaluation log

An evaluation log was maintained by the primary author (CD), who was also the primary evaluator of the program. Entries were made following interactions with the Memory Clinic to document evaluation processes and knowledge translation activities. All evaluation log entries followed the Objective, Reflective, Interpretive, and Decisional. (ORID) framework, a method for focused discussion presented in the business literature [32] that has been adapted to guide reflective journaling [33]. Each entry attended to the four ORID stages and included: (a) a description of the knowledge translation event including date and nature of the event, (b) evaluator reaction to the event, (c) interpretation and analysis of the event and (d) a description of how the KT event would guide future KT events. Log entries were entered directly and sequentially into a word processing document.

#### Program documents

Program documents included patient handouts, educational materials, program meeting minutes and final evaluation report.

### Data analyses

Pattern matching was used as the overall analytic strategy. This approach “compares an empirically based pattern with a predicted one” [21], where propositions are developed prior to data collection in order to identify a predicted pattern of variables. Propositions for this study were derived from the theoretical framework of Henry and Mark [18, 19] and informed by the knowledge translation literature.

1. Individuals who engage in a KT-informed evaluation will:
  - have a greater knowledge of assessments and interventions of memory disorders.
  - have a positive attitude towards research and evaluation.
  - refine clinical practices and process based on empirical evidence and evaluation results and processes.
2. Being engaged in a KT-informed evaluation will support program interactions and build knowledge translation capacity within the team.
3. The primary care organization will develop structures and practices to ensure data and evidence inform health service delivery and program development (i.e. use of electronic medical record to collect and use patient data).

Data were entered into an excel spreadsheet and tables were used to visually examine the data. Descriptive analyses were performed on the EROS [26] and CPAT [31]. Item averages were calculated for EROS [26] due to missing data, and subscale and total score averages were calculated for the CPAT [31]. Given the small sample size statistical significance was not calculated for either the EROS or CPAT. Qualitative interview data were digitally recorded and transcribed verbatim by a research assistant. Atlas ti, a qualitative data analysis and research software, was used to code data and identify themes. The primary author read all transcripts and a preliminary coding table was established. Transcripts were re-read, resulting in the collapse of ten codes, due to overlap. In total 20 codes were included in the final coding table from which seven broad themes emerged. Because of the small number of participants, quotes included in the manuscript are not identified by health profession.

A number of strategies were used to establish trustworthiness [34, 35]. Two transcripts were read and independently coded by a second investigator (LS) using the final coding structure. A second strategy to establish trustworthiness involved member checking. Participants were provided with interview summaries and asked to contact the primary author if any errors were noted, or if additional information should be included. None of the participants reported any errors or provided further information.

A third strategy involved triangulation of data methods, sources and investigators. The study included a number of data methods including interviews, questionnaires and program documents. Each contributed to the understanding of the influence of evaluation and how it can be used as a mechanism for IKT. Participants included members from a range of disciplines, who were both internal and external to the organization to provide different perspectives and experiences of participating in the evaluation. Finally, the investigation team was made up of two occupational therapists (CD, LL), one evaluation researcher and practitioner (LS), and one educational researcher (DK). The diversity of the team brought unique perspectives to the design, implementation and analyses and grounded the study in both research and practice.

## Results

**Fig. 1:**
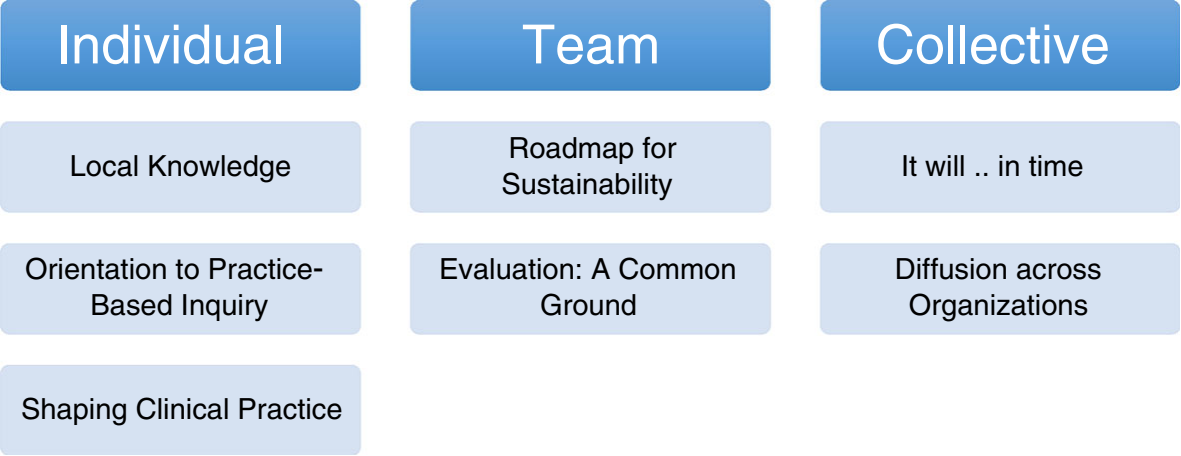
Evaluation for knowledge translation: themes and subthemes

There were a total of six members on the Evaluation Committee. Two members had worked on an interdisciplinary team for over 10 years, two members had worked between 5 and 10 years on an interprofessional team and two had worked on an interprofessional team for 1 year or less. The evaluation was found to influence the individuals, team and broader organizations in ways that were both intended and unintended. Seven overall themes were identified across the individual, interpersonal and collective levels. See Fig. 1 for an overview of the themes.

### Influence on the individual

#### Local knowledge

Individuals obtained knowledge from a range of both formal and informal sources. The sources of knowledge evolved over the course of the evaluation. Pre-evaluation questionnaires identified resources found within the Memory Clinic Training Manual as the most frequent source of formalized knowledge. Following the evaluation however, the weekly evaluation e-newsletter was identified as the source most frequently accessed for information. Virtual practice networks and online materials also provided important resources for participants.

Team members were a critical source of knowledge. During post-evaluation interviews, all but one of the members identified the team as the first place they would turn to for information. In addition to the immediate team, individuals from the Memory Clinic training team were also identified as key sources of information. In these situations, communication was primarily between similar disciplines, for example the nurse at the Memory Clinic would contact the nurse at the training site. Overall, when knowledge was local and research was considered within context it was seen as relevant and directly applicable. “I want the local, and the reliable [information], and a study from Toronto, from someone with who knows what credentials, isn’t any help to my clients that are here right now” (FpP7:8:46).

The intentional knowledge translation strategies used during the course of the evaluation [10], coupled with evaluation processes and emerging results provided the team with local practice-based knowledge.

“The evaluation informed my practice for sure, because not just the evidence-based approach and articles that [the evaluator] was sending, but also we have program objectives and knowing what our focus was informed me as well” (PostP1:1:4).

#### Orientation to practice based inquiry

While a KT-informed evaluation sought to sensitize individuals to research the Edmonton Research Orientation Scale (EROS) [26] did not demonstrate any shift in orientation towards research. EROS subscale scores could not be calculated due to missing data on a number of items; most notably within the Involvement in Research and Evidence Based Practice subscales. As a result the average rating per item was calculated (see Table 2). Item averages remained essentially the same across all four subscales, with two subscales slightly lower at follow-up and one slightly higher, suggesting the evaluation had minimal impact on the individual’s orientation towards research. Knowledge related to five aspects of research increased slightly from pre-to-post evaluation. There was no change in time spent reading or participating in research or research related activities.

**Table 2:** Edmonton Research Orientation Scale (EROS)

EROS subscale	Pre-evaluation (*n* = 5)^a^	Standard deviation	Post-evaluation (*n* = 6)^a^	Standard deviation
Valuing Research	3.7	0.7	3.7	0.6
Research Involvement	2.4	0.9	2.2	0.6
Being on the Leading Edge	3.8	0.8	3.9	0.5
Evidence Based Practice	3.7	0.6	3.5	0.7
Total:	3.4	0.9	3.3	0.9
Understanding Research Design	2.6	1.5	2.8	1.2
Statistics	2.6	1.1	2.8	1.2
Research articles in journals	3.4	1.1	3.7	0.9
Grant application procedures	1.8	0.8	1.8	0.9
Ethical review procedures	1.8	1.2	2.0	1.1

^a^item averages on a 5-point scale

While general research orientation, as measured by the EROS [26], remained unchanged, interview data highlighted the role evaluation played in making research more accessible.

“I think [the evaluation] humanized the idea of research instead of it being all the research out there that I am not part of, so this brought it into my realm of general practice and day to day practice” (postP4:4:1).

The evaluation served to orient clinicians to practice based inquiry, bridging the research-practice divide. “It seems so practical, it just seems so natural and I always saw research as more academic” (postP4:18:48). Through the evaluator’s presence and engagement in the evaluation, clinicians not only gained knowledge about the process of conducting an evaluation, but more specifically how knowledge created through evaluation translated to practice. “Having [the evaluator] so involved helped us learn more about what an evaluation is, what it looks like, how it works into the day-to-day stuff we are learning, and how it translates” (postP1:42:92).

The KT-informed evaluation sought to model sustainable practice based inquiry. While the evaluation did not appear to influence individuals’ orientation or attitude to research broadly, it supported an orientation to local practice-based inquiry and knowledge.

#### Shaping clinical practice

Changes to clinical practice were documented over the course of the evaluation and were related to both the evaluation processes and results. Not only did individuals gain knowledge during the evaluation they were receptive to making changes to practice as a result of this knowledge. “We have to be open to change what we find does need to be changed…you have to be willing to change” (postP3:6:30). Over the course of the 8-month evaluation a number of refinements were made to the assessment and intervention practices and Memory Clinic processes. Refer to Table 3 for description of changes that were made and how the evaluation process linked to these changes.

**Table 3:** Influence on clinical activities and processes

Program enhancements and modifications made throughout evaluation	
Clinic assessments	Evaluation activities implemented that supported program enhancements
1. Addition of a gait assessment into the assessment protocol.	Memory clinic network conference
	Weekly evaluation update - E-newsletter
	Memory clinic process meeting
2. Addition of vital statistics into the assessment protocol.	Memory clinic network conference
	Memory clinic process meeting
3. Additional of “Since we last saw you”, an assessment of community supports into the assessment protocol.	Evaluation process meeting
	Evaluation results – chart audits, patient and caregiver feedback surveys
Clinic intervention/follow-up activities	Evaluation activities implemented that supported program enhancements
1. Enhancement of educational materials (driving, enhanced mail-out package, educational binder)	Evaluation results - patient and caregiver feedback surveys
	Weekly evaluation update - E-newsletter
2. Patient action plans	Evaluation process meeting
	Evaluation results – patient and caregiver feedback surveys
3. Patient/caregiver Workshop: brain gym	Evaluation process meeting
	Evaluation results – patient and caregiver feedback surveys
4. Patient/caregiver workshop: dementia and diabetes	Evaluation process meeting
	Evaluation results – patient and caregiver feedback surveys
Memory clinic processes	Evaluation activities implemented that supported program enhancements
1. Patient services coordinator: new title and timing of introduction	Evaluation process meeting
	Evaluation results – patient and caregiver feedback surveys
2. Stopping of evaluation process meetings	Evaluation process meeting
4. Assessment summary forms (Under consideration at end of evaluation.	Evaluation process meeting
	Evaluation results – chart review
5. Timing of patient/family education	Evaluation process meeting
	Evaluation results – patient and caregiver feedback surveys
6. Patient chart scanned into EMR	Evaluation results - physician feedback survey

Three elements of the evaluation were seen to influence clinical practice; knowledge gained from engagement in the evaluation process, empirical evidence provided during the evaluation, and emerging evaluation results. Participant engagement in evaluation created a culture of learning and laid the foundation for knowledge translation. “When you see [the evaluation] and you’re involved in it, and doing it, it’s more hands on, it’s more practical, it’s apt to be more useful” (postP3:25). Similarly, evaluator engagement in the program supported knowledge translation.

“Having [evaluator] so involved has helped us learn about what an evaluation is and what it looks like, how it works into the day to day [information] we are learning and how it translates…[evaluator] being involved really helped us getting it and understanding it (postP1:42:92).

Fundamentally, the knowledge translation focus of the evaluation sought to support patient care “this evaluation…it is being done to produce better quality patient care and I think we all know that now” (postP1:41:90). Weekly e-newsletters, offered a source of empirical evidence upon which practitioners grounded their assessment practices.

Just knowing what is happening…the updates and some of the research articles…that guides me and that started the gait [assessment] process, so it helped us if we got stuck in our ways and gave us new ideas (postP2:2:4).

Interventions were also supported by the intentional knowledge translation activities of the evaluation.

What we developed here was a [patient education] binder…some tips about eating and exercise and all of that was pulled from the evidence based practice stuff I pulled from Dr. [X,] or things [the evaluator] sent us or things that the team provided that they found to be helpful (postP1:35:66).

The Memory Clinic team was particularly receptive to emerging data derived from patient and caregivers, which in turn had a strong influence on Memory Clinic processes and clinical practices. The patient focus was seen at the clinical level and many of the clinicians identified that patient interactions was the element of clinical practice most influenced by the emerging evaluation data. “I have learned to ask more open ended questions and dig deeper and get better detailed answers” (postP5:6:24). Another clinician reported “it has changed the way I do the testing and assessments, building that relationship” (postP6:9:24).

The Memory Clinic was part of a larger network of clinics and receiving the ongoing feedback from the emerging evaluation also gave individuals the confidence and the structure to refine their practice. It also gave clinicians confidence in their own clinical practice.

We kept refining the process based on the feedback, based on [the evaluation], refined it, refined it, refined it, and the whole collection of information from the patients, and how we recognize that, and how we record that, and access it later. We are more confident (postP4:21:56).

Feedback also supported changes to program delivery. “So, once we got that feedback… that changed how we were thinking about educating people and the timing of the education” (postP1:36:68). Changes were also made to administrative processes based on feedback “We kept changing our forms and making them better” (postP2:10:28).

Participants reported an increased use of memory related assessments and interventions over the course of the evaluation. Individuals (*n* = 5) reported using an average of 3 assessments (range1-5) on the pre-evaluation Memory Disorders Knowledge Questionnaire, compared with an average of 8 (range 4 to 17) assessments after the evaluation. The same trend was observed for interventions. Individuals (*n* = 5) reported using an average of 2 (range 0 to 3) interventions when working with individuals with memory disorders before the evaluation and an average of 5 (range 3 to 8) interventions on the post-evaluation questionnaire. Referral to community supports was not identified as an intervention strategy on the pre-evaluation questionnaire, whereas all but one of the respondents on the post-evaluation questionnaire reported accessing community resources for patients and their families/caregivers.

### Influence on the interpersonal

#### Roadmap for sustainability

With the exception of the early addition of a community pharmacist and physician, team membership remained stable over the course of the 8-month evaluation. However, as the evaluation was concluding the team underwent substantial personnel changes, including two members going on maternity leave, the addition of another pharmacist and two members leaving the primary care clinic; including the Executive Director. Only 1 month after the evaluation was completed, five new members had joined the team, representing a 62 % turnover rate.

As membership changed, so too did the teams knowledge that was co-created over the course of evaluation. Prior to the changes in personnel “We were all sitting in that room together, so I know that information that I got from you…and you heard what I got…we all heard” (fpP7:19:104). However, as new members entered “we don’t really know what everyone else [knew]” (fpP7:19:100).

Despite new membership there was a commitment to sustaining the team’s clinical knowledge base and building on the evaluation “we want to keep learning” (postP5:11:63). One of the original team members informally took on the responsibility of creating strategies and mechanisms to transfer knowledge to the new members, passing along “the essential building blocks of this clinic and [handing] them out to everyone…so you have got a pillar who continues” (fpP4:4:26). Strategies included laminating summaries of memory disorder assessments and the development of a memory disorder clinical reasoning flowchart.

Supporting the team’s informal KT leader, were formalized tools that provided a roadmap for the team. “None of us could do this alone, so many people have given us the tools…[XX] just making sure the tools are handed down in their original, authentic form” (fpP4:6:54). There were clear supports to translate clinical knowledge however there was less evidence that structures were in place to support ongoing learning through evaluation. On one hand the evaluation was seen as one tool within the KT toolkit, offering processes to both collect data and provide ongoing feedback to the team. “[the evaluator] has given us clinical applications that actually will guide what we do” (fpP4:7:66). On the other hand, no formal procedures were in place to facilitate data collection and reporting. Unlike with the clinical knowledge, no team member had informally stepped into the role to translation evaluation knowledge or practices.

#### Evaluation: a common ground

The evaluation of the Memory Clinic began during the early formation of the team, and prior to the start of the implementation of the clinic. The evaluation provided a common ground for the team members, all of whom came from different disciplinary backgrounds. Through the participatory processes of the evaluation, the team developed program goals and objectives. This process had a number of benefits to the team. First it encouraged the team to shed their disciplinary focus.

“Hearing what kind of things people said for goals, it was not what I expected. From the doctor, I would have expected it would be to give a clearer diagnosis. But instead it was to support the client and the caregiver” (postP2:28:77).

Second, the program goals and objectives served to centre the team and pull members towards a common focus. “Those first few meetings trying to take the objectives and keep them in mind…and just to have clear objectives that we shared” (postP4:21:56).

The emerging evaluation findings offered program based knowledge, which crossed disciplinary boundaries. Team members were required to make sense of how the information influenced the team as a whole and then their individual practices. One team member reflected on how the emerging evaluation results heightened her awareness of the need to strengthen collaborative practice.

“It heightened my awareness of caregiver burnout, the need for the services here… the need to find strong partnerships especially with the Alzheimer’s society and working side by side…and somehow being more mindful of collaborative practice” (postP1:11:28).

The team viewed the Memory Clinic as a model of interprofessional collaboration in the primary care clinic. “It would be great if our other programs were run like that” (postP6:12:30). Because of the commitment to the team, there was also a commitment to the evaluation.

“I think the fact that the Memory Clinic is new and an exemplar of interprofessional collaboration within the [primary care clinic] creates a deeper commitment to both the evaluation and openness to dementia research and networks” (Evaluation log, September 13, 2012).

Results of the Collaborative Practice Assessment Tool (CPAT) [31] further demonstrated the team’s collaboration. All domains of the CPAT scores increased over the course of the evaluation, with a total CPAT score before the evaluation of 321 and 362 following the evaluation (Table 4).

**Table 4:** Collaborative Practice Assessment Tool (CPAT)

CPAT subscale (total subscale score)	Pre-evaluation (*n* = 6)	Standard deviation	Post-evaluation (*n* = 6)	Standard deviation
Mission (56)	49	3.5	52	2.8
Relationships (56)	52	3.6	55	2.6
Leadership (63)	50	5.7	55	9.0
Roles (70)	58	9.5	64	8.7
Communication (48)	34	5.5	41	4.2
Community (28)	24	1.7	27	2.0
Conflict (42)	26	2.7	37	4.4
Patient involvement (35)	28	4.1	31	5.0
Total (398)	321	20.5	362	20.8

Conflict and communication were the two domains that demonstrated the greatest change scores. The results of the opened ended questions reiterated communication as a team strength as well as the establishment of a culture of collaboration, involving trust, respect and openness to others ideas. The team created a term to capture the collaborative spirit they felt. “Teamy, we call it” (fpP4:).

### Influence on the collective

#### It will… in time

There was little evidence to suggest that the primary care organization in which the Memory Clinic was situated was influenced by the evaluation. While the evaluation was seen as foundational to the development of the Memory Clinic, the influence on the primary care clinic was not felt. “I just don’t know if it has trickled down to the broader health team…I think it will in time, and I think that as other programs evolve. I think it is foundational” (fpP3:26:118). No formal evaluation structures were in place for any other programs and there were no reported plans to formally introduce evaluation to other programs.

Over the course of the evaluation, no changes regarding the use of the electronic medical record (EMR) for evaluation purposes were reported. Both before and after the evaluation the EMR was primarily used for patient booking, charting and communication purposes. “It is just for recording and booking” (preP1:1:2). “To get a medical history and see what other people have done with the client…and then we use more the messaging for the referrals” (preP2:14:40). However there was an acknowledgement that the EMR could facilitate ongoing evaluation. “I think that it is the next step. I think we have the paper end of things…now we need to put that over to the EMR” (postP3:17:80). From an administration perspective, the EMR was also used for statistical purposes to identify numbers of patients and to obtain targeted outcomes for mandated reporting; not as a means to inform practice.

The open-ended responses on the CPAT [31] suggested an overall lack of communication between the Memory Clinic and broader primary care organization, which included 7 physicians, a dietician, a nurse practitioner, and a respiratory therapist. Post-evaluation CPAT results identified the need for collaboration with the broader clinic as the most important area of improvement within their own team. Additionally, communication to the broader primary care clinic was identified as a challenge to the team’s own collaboration. Given the lack of communication at a clinical level, it is not surprising that the evaluation did not exert an influence on the primary care organization.

#### Diffusion across organizations

Despite the lack of influence on the immediate organization, its unintended influence was demonstrated in two external organizations. In the first case, one of the members of the Memory Clinic team, who represented a community agency, described how the evaluation changed how she would collect data to inform her practice. As a result, new data collection mechanisms were created that subsequently altered the practices of another individual within the second organization. “I learned more about how I might be able to collect that type of information…I was able to give that information to my co-worker…and then he went and changed [how he collected information]” (fpP1:21:114).

In the second case, the Executive Director of the primary care organization became a manager at a new health organization and brought evaluative thinking with her. The implications of this experience laid the foundation for thinking about how evaluation might be embedded into the new organization. “Evaluation; we have to…build a framework or some sort of guidelines for every program that we do… there is an evaluation component” (fpP2:1:2).

So while the capacity of the original primary health organization did not appear to be enhanced within the time frame of this study, individuals who were involved began to see themselves as having a responsibility for carrying over what they had learned through evaluative inquiry into their new settings.

## Discussion

Ultimately this study sought to encourage more expansive thinking about how evaluation can be used in primary care to bridge the evidence to practice gaps. The study provides evidence that evaluation processes and results can influence health care practices in primary care.

### Supporting practice-based knowledge

At the level of the individual, participating in a participatory evaluation designed to support KT influenced individuals’ knowledge about the program, attitudes towards practice-based knowledge and clinical practices and processes. Both the emerging evaluation results and activities, including weekly e-newsletters, were important sources of knowledge. The study clearly supports the literature that has found primary care clinicians rely heavily on practice-based tacit knowledge and colleagues [1, 36]. An ethnographic study exploring decision making of primary care clinicians, found clinicians rarely accessed, appraised, and used explicit evidence directly from research or other formal sources [37]. Instead, the authors describe the use of ‘mindlines’; internalized tacit guidelines, in part informed by brief reading, but primarily informed by their interactions with each other, with opinion leaders, and by other sources of largely tacit knowledge. Mindlines are built on early training, their own and their colleagues’ experience and reinforced by the collective practice. The evaluation process was particularly congruent with the notion of building mindlines through its emphasis on practice-based knowledge, integration of context sensitive research, and a participatory approach that offered opportunities to interact and engage in the process of inquiry.

Clinicians’ self-reported orientation and participation in research did not change over the 8-month evaluation. A few factors may have contributed to this. Questions on the EROS [26] relate specifically to research and do not use broader terms of inquiry or evaluation to which the clinicians may have more readily related. As well, the Memory Clinic makes up only a small portion of clinicians’ roles within the primary care organization and therefore the evaluation of the Memory Clinic may not have been influential enough to tip the clinicians’ orientation or attitude towards research. The results provided further evidence however that primary care clinicians are still not oriented toward research. Evaluation has been described as ideally situated to bridge the research-practice divide [38]. The evaluation facilitated a positive attitude towards practice-based inquiry and the learning that occurs in this process. There is a growing body of literature on the role of evaluation in supporting both individual and organizational learning [39–41]. This study lends further support to this work and highlights evaluations important role in knowledge exchange in primary care.

Patient and caregiver feedback data appeared to have the greatest influence on practice behaviors and was the impetus for many of the ongoing program refinements. This is an interesting finding and offers a more fine-grained understanding as to the sources of data that may be most influential to clinical behaviors. Christie [42] used the Pathways of Influence [19, 20] to examine influence of evaluation data on decision makers actions. Christie [42] found that large scale and case study data were most influential, suggesting that different contexts and stakeholders attend to data in different ways. In a primary care setting, evaluation processes and results may be most influential when there is a clear link to patient services and outcomes. This study did not include patients or families on the evaluation committee however these results suggest that including this stakeholder perspective could further sensitize the team to patient data.

A fundamental goal of KT is to “improve the health of Canadians” [3]. This study explored the behaviors of the clinicians, but did not examine how the evaluation influenced patient and family outcomes. Because the evaluation began prior to the start of the program there was no baseline data upon which to measure changes in patient outcomes. Further research that includes patient outcomes is required to more fully explore the role evaluation to support KT.

### Interpersonal

The evaluation was seen to influence interpersonal behaviors through the development of social norms. Research on interprofessional collaboration in primary care has identified the development of common patient goals as an important indicator of team function [43, 44]. The results of the study suggest that an evaluation can also provide a common goal and focus for a primary care team. The evaluation was seen to influence the team’s social norms, supporting the team in thinking beyond their disciplinary boundaries and develop a shared vision and common language. No other studies could be found that examined the influence of evaluation on interprofessional primary care practice. With increasing focus on interprofessional models of primary care and emphasis on quality improvement initiatives it is important to understand how evaluation can support collaboration and is an area that warrants further exploration. Despite the small participant numbers, a significant difference was found on five of the eight CPAT [31] subscales, suggesting that the CPAT [31] could be a potentially powerful tool to examine interprofessional collaboration.

While the evaluation helped establish common program goals and objectives, 3 months after the evaluation ended the team was almost entirely new. Turnover of program personnel is common within health care and an important element to consider in any KT study. A study conducted in a hospital environment reported 1-year turnover rates of 49 % for allied health, 29 % for nurses and 9 % for physicians [45]. Woltmann and colleagues [46] examined the impact of turnover on evidence-based practices. Seventy one per cent of respondents reported that turnover influenced the implementation of the evidence-based guidelines [46].

Within the current study the team had developed clear strategies to translate clinical knowledge to new members. There was less evidence of strategies to support the ongoing use of program-based evidence, or the translation of evaluation knowledge to new members. This finding has a number of potential implications for future evaluations. Looking back to the three dimensions of participatory evaluation [24], the evaluator led the overall evaluation with input from the team. While this ensured the ongoing implementation of processes to support learning and knowledge exchange during the evaluation, it did not adequately consider the maintenance of these after its completion. In other words the long-term influence of the evaluation to support ongoing knowledge translation appeared limited. The results suggest that divesting control, or a graded approach, where the evaluator fades out over time, might enable the evaluation to have greater long-term influence. However, further research needs to be completed to determine the longer-term impact of participating in evaluation and what elements and activities could help support long-term influence.

The fact that one individual led the strategies to translate clinical knowledge, suggests that the influence of one person should not be underestimated. Garcia-Irarte, and colleagues [47] have described how one individual served as an effective catalyst for building evaluation capacity within a community based organization. Similarly, a systematic review found opinion leaders, both alone or combined with other strategies, were effective in promoting evidence-based practice [48]. These studies, as well as results from the current study suggest that a KT-informed evaluation also needs a dedicated leader. Primary care organizations should consider formally identifying an individual or role within the team to translate evaluation knowledge and facilitate processes that support evaluation as an ongoing form of KT within primary care.

### Collective

The study found the evaluation did not have any immediate influence on the primary care organization in which the Memory Clinic was situated. To some extent this is contrary to what would be expected based on the growing body of literature on evaluation capacity building. Evaluation capacity building (ECB) is described as the “intentional work to continuously create and sustain overall organizational processes that make quality evaluation and its uses routine in organizations” [49]. A recent systematic review of the ECB literature found that 92 % of evaluations reviewed produced changes at the level of the individual and 77 % demonstrated organizational level changes [50].

The Pathways of Influence model [18, 19] can help to explain these findings. The intentional evaluation activities were focused on supporting KT at the level of the individuals and interpersonal, suggesting that evaluation processes and activities are most likely to influence the level at which they are targeted. In other words, building individual knowledge does not appear to directly influence the collective. The KT literature has largely described interventions that have targeted individuals and there is increasing attention being paid to organizational level interventions [51]. As evaluations have been shown to support organizational learning, future evaluations are encouraged to include activities that specifically target the organization.

### Policy implications – continuous quality improvement

Continuous quality improvement (CQI) is one of the key elements of primary care reforms in both Canada and abroad [52]. The findings of this research can provide important insights into CQI initiatives in primary care. In the province of Ontario, Canada, where this research was conducted, a number of recent policy initiatives and documents [53, 54] create a context that is ideally situated to receive and potentially implement recommendations from this research. Not only have recent provincial health policy documents viewed primary care as the “natural anchor for patients in our health care system” ([53] p. 8), but there is a provincial goal to “expand our focus on quality improvement to family health care, and ensure that all family health care providers are equipped to integrate the latest evidence-based care into their practice” ([53], p. 9). A specific organization dedicated to supporting quality improvement activities, Health Quality Ontario, has been established by the provincial government [55] and yearly documentation is submitted by all interprofessional primary are teams to support quality improvement. While there is infrastructure to support CQI documentation, there are no processes to: 1) engage primary care providers in quality improvement initiatives, 2) provide findings to clinicians in a clinically meaningful way, or 3) link this data to evidence-based practice.

This research suggests that not only can evaluation provide program specific knowledge to support ongoing professional learning and program improvements, but highlights that strategies and activities can be intentionally included to support learning. Specifically CQI could: (a) include activities to promote individual and organizational learning so CQI activities are meaningfully imbedded into practice, (b) ensure the CQI process is collaborative in nature and inclusive of interprofessional perspectives, (c) embed opportunities for discussion and reflection so teams can make meaning of the data, and (d) use the CQI to identify relevant research and knowledge networks to further support learning. This study also suggests there is a need to either intentionally build capacity of individuals, teams and organizations to facilitate CQI within primary care, or to provide financial or human resources to support the ongoing learning and improvement process.

### Future research

The case study method provided an in-depth look at one evaluation designed to support KT and while it provides insights into the role and potential of evaluation, the results cannot be generalized broadly. It is anticipated that additional sites could provide further insights into the influence of evaluation in supporting knowledge exchange. Given that the study only included follow-up at 3-months, it would be of interest to examine the long-term influence of a KT-informed evaluation one to two years after the evaluation. Intentional KT activities were focused on the individuals and team. Future work is required that includes activities targeted at building KT capacity of the organization. Finally, additional studies should consider the influence of a KT-informed evaluation on patient outcomes.

## Conclusion

This study provides the first known exploration how evaluation can support KT in primary care. Evaluation is a fundamental component in building quality primary care and is ideally situated to support individual, team and organizational learning by offering an accessible, applicable and relevant form of KT.

This research identified a number of strategies, structures and approaches that supported KT throughout the evaluation.

1. A participatory approach is a basis requirement for any evaluation designed to support KT and congruent with recommendation of adopting an IKT approach in primary care. Engagement of stakeholders should be as deep as is feasible within the program’s context in order to support learning and knowledge translation.
2. The evaluator needs to have sustained and deep interaction with the program. Doing so provides an understanding of (a) the program and its processes, (b) the types of knowledge that is valued and used, (c) the format in which knowledge is best received, (d) team interactions, (e) organizational culture.
3. The evaluator must capitalize on the knowledge-brokering role of the community stakeholders, providing them with opportunities and structures to both bring knowledge into the program, and share program knowledge with the broader community.
4. The evaluator needs to commit to gaining a strong understanding of the empirical literature that grounds the program. Not only does this provide credibility, but provides the foundation to ensure that relevant and contextual empirical evidence is woven throughout the evaluation.
5. The evaluator needs to gain an understanding of the broader knowledge networks that can inform the program. Many knowledge networks function as online communities and therefore the ability to navigate and critique online resources is required.
6. In order to support ongoing refinements to practice there needs to be frequent and ongoing communication of both emerging evaluation results and relevant empirical evidence and resources. The evaluator must be sensitive to the frequency of communication, so as not to overwhelm the program.
7. The evaluator needs to build in opportunities for the program to engage in conversation around emerging evaluation results and actively support knowledge exchange. For example, regular meetings that focus on meaningful program data and attend specifically to patient feedback and data will likely enhance the integration of this knowledge into practice.
8. The evaluator must be intentional in building the capacity of individuals, the team and the organization. Findings from this research show that activities must targeted both the individual and organization to ensure the KT-informed evaluation exerts an influence at each of these levels.

## Additional file

Additional file 1: Memory Clinic Knowledge Questionnaire. Word Document (DOCX 16 kb)

## Abbreviations

CPAT: Collaborative practice assessment tool
ECB: Evaluation capacity building
EROS: Edmonton research orientation survey
IKT: Integrated knowledge translation
KT: Knowledge translation
ORID framework: Objective, reflective, interpretive, and decisional framework
