# Supplementary material for: Using program evaluation to support knowledge translation in an interprofessional primary care team: a case study
Source: BMC Fam Pract. 2016 Oct 6;17:142. doi: 10.1186/s12875-016-0538-4 (PMC5053347; doi:10.1186/s12875-016-0538-4)
Supplement: Additional file 1: — Memory Clinic Knowledge Questionnaire. Word Document (DOCX 16 kb) [file 12875_2016_538_MOESM1_ESM.docx]

Memory Clinical Knowledge Questionnaire

1. I have worked with individuals with memory disorders for:

< 1 year 1-5 years 5-10 years >10 years

2. I am confident in my knowledge of memory disorders.

Disagree Somewhat Disagree Somewhat Agree Agree

3. I am aware of the current literature in my field related to assessment of memory disorders.

Disagree Somewhat Disagree Somewhat Agree Agree

4. If I needed to find current literature about memory disorders I would know where to find this.

Disagree Somewhat Disagree Somewhat Agree Agree

5. Please describe your current sources of information you use to inform your knowledge of memory disorders.

6. Is this typical of the sources of information you use to inform the rest of your practice?

Yes No

7. Please describe the current assessment you use when working with individuals with memory disorders:

8. I am aware of the current literature in my field related to the interventions for memory disorders.

Disagree Somewhat Disagree Somewhat Agree Agree

9. Please describe the current interventions you use when working with individuals with memory disorders:
